# *Eucalyptus gunnii* and *Eucalyptus pulverulenta *‘Baby Blue’ Essential Oils as Potential Natural Herbicides

**DOI:** 10.3390/molecules26216749

**Published:** 2021-11-08

**Authors:** Cristina Danna, Laura Cornara, Antonella Smeriglio, Domenico Trombetta, Giuseppe Amato, Pierluca Aicardi, Laura De Martino, Vincenzo De Feo, Lucia Caputo

**Affiliations:** 1 Department of Earth, Environment and Life Sciences, University of Genova, 16132 Genova, Italy; cristina.danna@edu.unige.it (C.D.); laura.cornara@unige.it (L.C.); 2 Department of Chemical, Biological, Pharmaceutical and Environmental Sciences, University of Messina, 98166 Messina, Italy; asmeriglio@unime.it; 3 Department of Pharmacy, University of Salerno, 84084 Fisciano, Italy; giuseppe.amato119@gmail.com (G.A.); defeo@unisa.it (V.D.F.); lcaputo@unisa.it (L.C.); 4 Pierluca Aicardi, Coldiretti Savona, 17100 Savona, Italy; pierluca.aicardi@coldiretti.it

**Keywords:** *Eucalyptus*, oil cavities, micromorphology, essential oils, natural herbicides, phytotoxicity, α-amylase, eco-compatibility

## Abstract

The phytotoxicity and eco-compatibility of essential oils (EOs) from *Eucalyptus gunnii* (EG) and *E. pulverulenta* ‘Baby Blue’ (EP), cultivated in Italy for their cut foliage, were investigated. Leaf micromorphology, EOs phytochemical characterization, and phytotoxicity were analysed. EP revealed a significantly higher oil gland density and a higher EO yield with respect to EG. In both EOs, 1,8-cineole was the major compound (~75%), followed by α-pinene in EG (13.1%) and eugenol in EP (7.5%). EO phytotoxicity was tested on both weeds (*Lolium multiflorum*, *Portulaca oleracea*) and crops (*Raphanus sativus*, *Lactuca sativa*, *Lepidium sativum*, *Solanum lycopersicum*, *Pisum sativum, Cucumis sativus*). EG EO inhibited germination of *P. oleracea*, *R. sativus*, and *S. lycopersicum* seeds (ranging from 61.5 to 94.6% for the higher dose used), while affecting only radical elongation in *S. lycopersicum* (ranging from 66.7 to 82.6%). EP EO inhibited germination of *P. oleracea* and *R. sativus* (ranging from 41.3 to 74.7%) and affected radical elongation of *L. sativum* and *L. multiflorum* (ranging from 57.4 to 76.0%). None of the EOs affected the germination and radical growing of *L. sativa*, *P. sativum*, and *C. sativus*. Moreover, EP EO was more active than EG EO in inhibiting α-amylase, a key enzyme for seed growth regulation. Brine shrimp lethality assay showed that both EOs are safe for aquatic organisms, suggesting their high eco-compatibility. The data collected provide useful information for future applications of these EOs in agriculture as safe and selective bioherbicides.

## 1. Introduction

Native from Australia, the genus *Eucalyptus* (Myrtaceae) contains about 800 species and is largely cultivated around the world in commercial plantations for several applications, such as cellulose, pulp, gum, essential oils, and honey production, as well as for construction and as ornamental plants [1,2].

In Italy, several *Eucalyptus* species were introduced at the beginning of the 1800s by plant collectors to be used as ornamental trees in private gardens [3]. During the early 1900s, these trees became largely cultivated, especially in South Italy, for reclaiming swampy lands and eliminating the outbreak of malaria [4]. Today, the *Eucalyptus* genus is still cultivated in many Italian regions, growing until 350 m above sea level. Long-established eucalyptus cut foliage plantations occur in Imperia and Western Liguria (Italy), in the Alpes Maritime (France), and in Cornwall (England), while less extensive plantations are also present in Ireland [5]. Nowadays, Italy is the leader in Europe in terms of the production and export of eucalyptus cut foliage, which is mainly sold on the Northern European market, where fresh and dried branches are used in floral compositions [6].

In Liguria, different species are cultivated for this purpose, such as *E. cinerea* F. Muell. ex. Benth., *E. gunnii* Hook.f., *E. pulverulenta* Sims cv ‘Baby blue’, and *E. parvula* L.A.S. Johnson and K.D.Hill. Correct pruning is useful to produce high quality juvenile stems from *Eucalyptus* species [7]. During harvesting, stems are carefully selected for the quality of branches based on the color and shape of the foliage. As a result, a large amount of waste biomass is produced. It is estimated that, in the Province of Savona, Western Liguria, where these plantations have an extension of 100 ha, a total of about 1000 tons per year of cut foliage is produced, with waste of more than 4% (4 tons), which is currently buried or burnt (Coldiretti, Savona; personal communication).

However, this discarded plant material is still rich in bioactive compounds with interesting biological properties. Recent studies have shown the potential of remnants from *Eucalyptus* pruning for transformation into added value products [8,9]. The genus *Eucalyptus* is characterized by the presence of many secondary compounds, such as phenolics, essential oils, waxes, and resins. The abundance of bioactive compounds suggests different applications, such as pharmaceutics, cosmetics, natural pesticides, insecticides, and herbicides [10].

The use of synthetic compounds for pest control has harmed the environment and human health. Glyphosate-based formulations, extensively used as herbicide worldwide, have been recently referred to as ‘probably carcinogenic’ [11]. For this reason, together with the development of resistance in weeds and pathogens, many countries have recently restricted or banned the use of such formulations [12]. Therefore, essential oils (EOs) showing bioherbicidal potential and not being persistent in the environment represent a valuable alternative to synthetic chemicals in integrated weed management. This possibility is of particular interest also considering that, in *Eucalyptus* plantations, the ground is still maintained weed-free using glyphosates, such as Roundap (Bayer). *Eucalyptus* EOs, rich in 1,8-cineole, can play an important role in modern agriculture to reduce the use of harmful herbicides and pesticides thanks to their allelopathic effect on weeds and to the interaction with noxious insects [13,14,15].

Phytotoxic effects of different *Eucalyptus* EOs have been reported on many common weed species. *E. globulus* EO showed significant effects on seed germination and seedling growth of *Amaranthus blitoides, Cynodon dactylon* [16,17], and *Chenopodium album* [18]. One of the main components of *Eucalyptus* EOs, eugenol, significantly inhibited the germination of *Cassia occidentalis* and *Bidens pilosa* at a very low concentration [19]. *E. citriodora* EO significantly affected both the radical and hypocotyl development of *Lolium multiflorum* [20], while *E. camaldulensis* EO completely inhibited seed germination of *Amaranthus hybridus* and *Portulaca oleracea* [21]. However, these EOs can also cause damage to some crops, making it essential to assess their selectivity [22,23]. The phytotoxicity on weeds is probably due to the alteration of several biochemical and physiological processes, affecting seed germination and/or hypocotyl and radical elongation [19,20,24,25]. These activities depend on the quantitative and qualitative composition of the EO of each individual *Eucalyptus* species. Therefore, the identification of the species source of EOs is important, especially when pruning material comes from mixed plantations, where different species are found side by side. Micro-morphological analyses are an essential starting point of quality control, especially for plant remnants and by-products [26]. These analyses involve the use of light microscopy (LM) and scanning electron microscopy (SEM), allowing to characterize the plant material, highlight contaminations [27], and establish the botanical identity.

We carried out the micro-morphological characterization of pruning remnants from two different *Eucalyptus* species, namely *E. gunnii* and *E. pulverulenta* ‘Baby blue’, cultivated for cut foliage in the hinterland of Western Liguria (Italy). In addition, the phytochemical profile of the EOs obtained from the biomass of these plants was analyzed and tested for selective phytotoxicity on weeds and crop seeds. Finally, their eco-compatibility was evaluated for safe applications in agriculture as bioherbicides, according to the principles of a circular economy.

## 2. Results and Discussion

### 2.1. Micromorphological Studies

**Figure 1:**
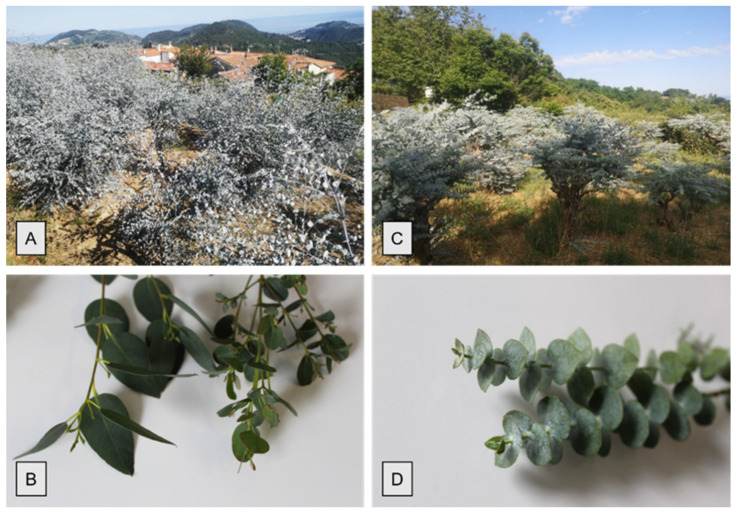
Plantation and branches of *E. gunnii* (**A**,**B**) and of *E. pulverulenta* ‘Baby blue’ (**C**,**D**).

Micromorphological analyses were performed on juvenile leaves of *E. gunnii* (EG) and *E. pulverulenta* ‘Baby blue’ (EP) collected during January 2021 in the hinterland of Western Liguria (Italy) (Figure 1A–D). EG shows dark green leaves, varying from sessile and rounded to peduncolate and more elongated (Figure 1B), while the EP blue-gray leaves are sessile with a rounded shape (Figure 1C).

**Figure 2:**
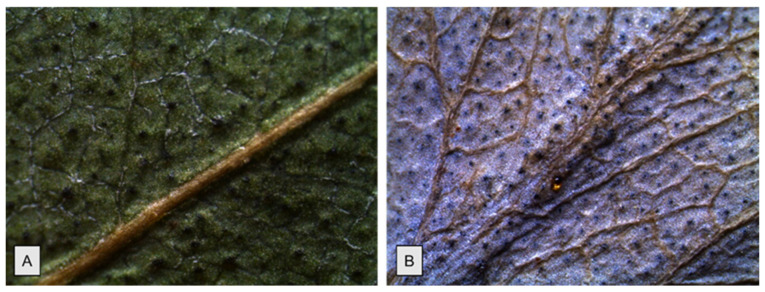
Abaxial surfaces of *E. gunnii* (**A**) and of *E. pulverulenta* ‘Baby blue’ (**B**) at 2.5× magnification.

The leaf surface of both species is glabrous, leathery, and waxy and shows many oil glands, giving them a dotted appearance (Figure 2). The venation pinnate-reticulate is slightly evident in EG (Figure 2A), while it appears much more prominent in EP (Figure 2B). Similarly, a larger wax deposition results in a more intense powdery blue-grey surface in EP (Figure 2B).

**Figure 3:**
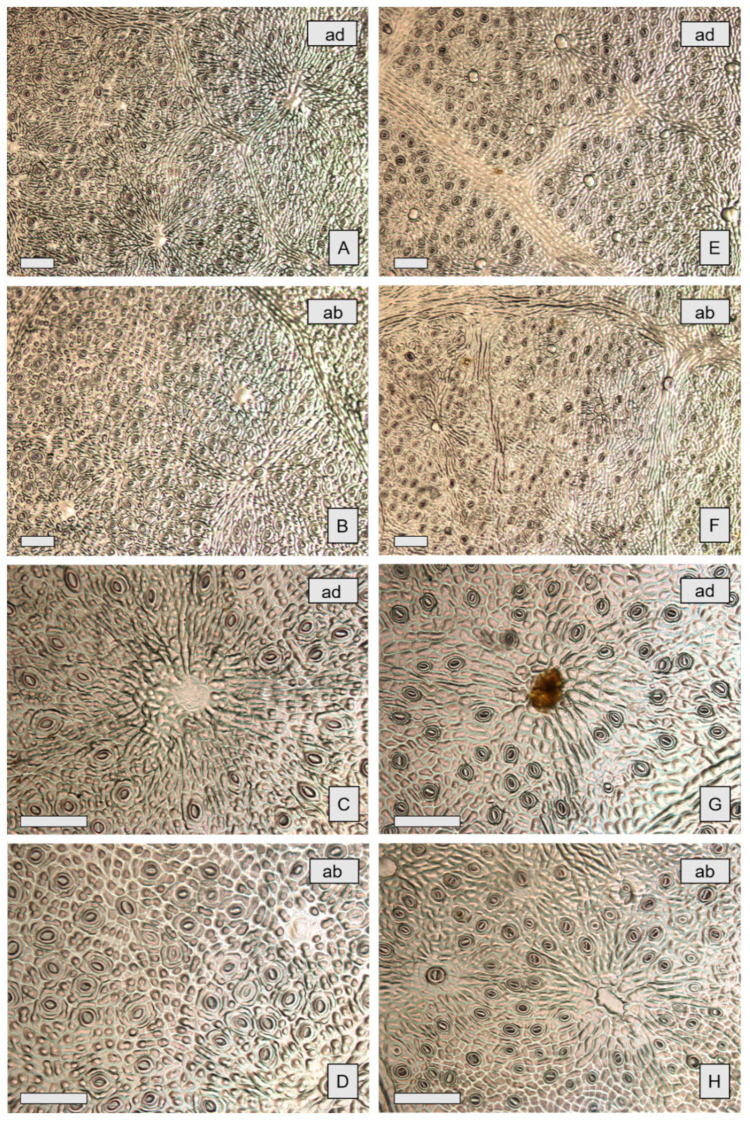
LM—**A**–**D**: *E. gunnii* (EG); **E**–**H**: *E. pulverulenta* ‘Baby blue’ (EP). Comparison between leaf peelings from abaxial (ab) and adaxial surfaces (ad), at different magnifications. EG: adaxial surface at 10× (**A**) and at 20× (**C**); abaxial surface at 10× (**B**) and at 20× (**D**). EP: adaxial surface at 10× (**E**) and at 20× (**G**); abaxial surface at 10× (**F**) and at 20× (**H**). Scale bars: A–H = 100 μm.

**Figure 4:**
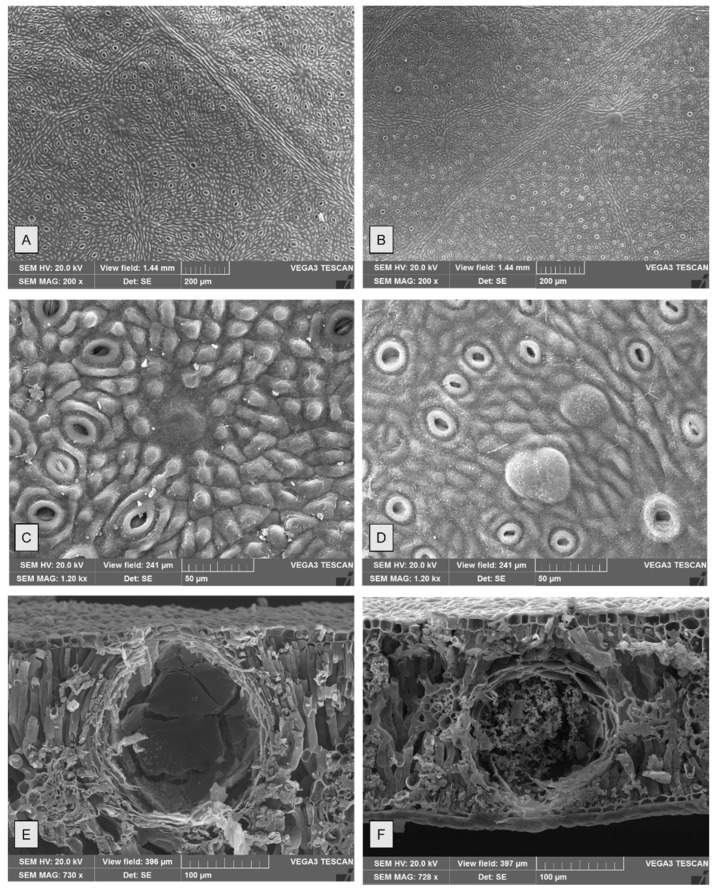
SEM—Comparison between *E. gunnii* (**A**,**C**,**E**) and *E. pulverulenta* ‘Baby blue’ (**B**,**D**,**F**) leaves. Adaxial surfaces at 200× (**A**,**B**). Overlying cells surrounding oil cavities in the abaxial surfaces, at 1200× (**C**,**D**). Leaf transversal sections showing oil cavities, containing amorphous material (**E**,**F**).

The observation by both light microscopy (LM) and scanning electron microscopy (SEM) showed in both species the amphistomatal leaves with anomocytic stomata, oval or rounded, appearing at the same level as the neighbouring epidermal cells and as the overlying cells surrounding oil cavities (Figure 3A–H and Figure 4A–D). These features agree with data previously reported for other *Eucalyptus* species [28,29]. Trichomes are absent, as also reported for *E. cinerea* F. Muell. ex Benth. by Soliman and coworkers [30].

Epidermal peelings showed amorphous yellow oil content, sometimes spread out among the cavities (Figure 3G). Epidermal cells in both species are polygonal isodiametric or slightly elongated in shape. Numerous papillae were observed in EG (Figure 4C), while they were not visible in EP (Figure 4D). Significant differences in glands’ density were found among the two species, with EP showing a higher mean gland density (Table 1). In addition, in each species, significant differences in gland density were also found between adaxial and abaxial surfaces of the leaf (Figure 3A,B,E–F and Figure 4A,B), as shown in Table 1.

Similar data on oil gland density have been reported for another species, namely *E. polybractea* F. Muell. ex R.T. Baker [31]. However, the number and the size of oil glands vary among the species and, in some cases, it is very high, such as in *E. protensa*, showing up to 3400 oil glands cm^−2^ [32].

**Figure 5:**
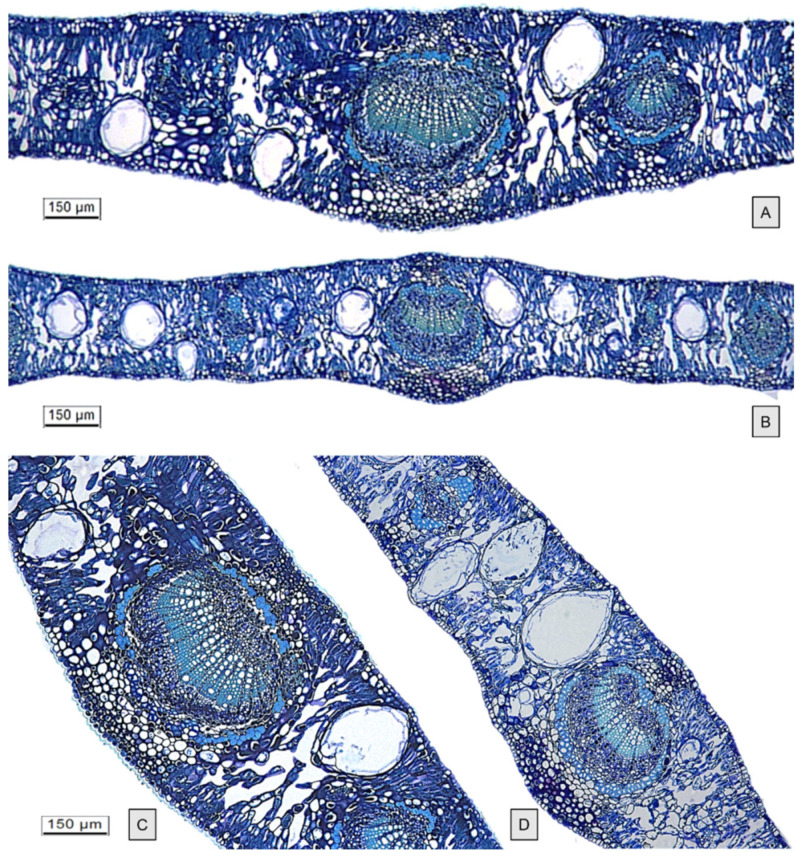
LM—TBO staining. Transverse sections through leaf midrib of *E. gunnii* (EG) (**A**–**C**) and *E. pulverulenta* ‘Baby blue’ (EP) (**B**–**D**), showing oil cavities varying from spherical/ellipsoidal to pear-shaped.

In leaf transversal sections (Figure 5), the oil glands appear distributed throughout the mesophyll and are more protruding in the upper surface in both species. The oil cavities cut close to the gland midpoint appeared spherical/ellipsoidal or pear-shaped. No significant differences were found between gland dimensions of the two species (Table 1). Each gland had a single internal epithelial layer, and the amorphous content was visible within the cavity (Figure 4E,F and Figure 5C,D).

### 2.2. Chemical Composition of Essential Oils

**Figure 6:**
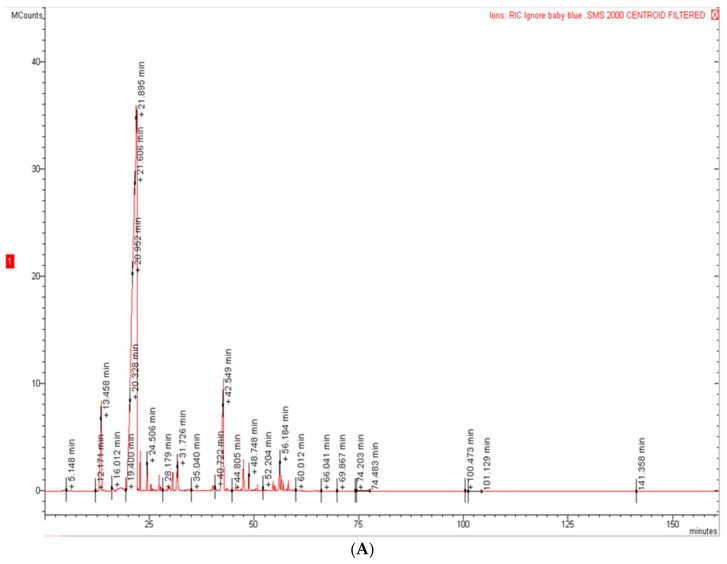

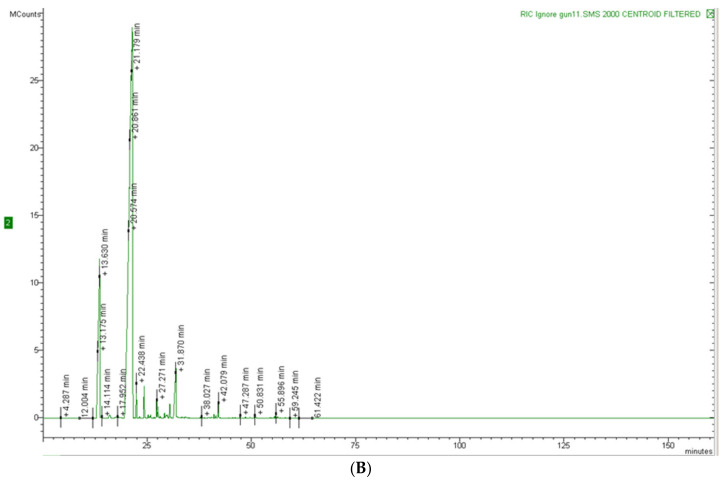
GC chromatograms of *E. pulverulenta* ‘Baby blue’ (EP) (**A**) and *E. gunnii* (EG) (**B**) EOs.

The hydrodistillation of the aerial parts of EG and EP furnished pale yellow EOs at a yield of 1.1 and 1.6% on a dry mass basis, respectively. The yield of the two EOs is an important parameter to consider, because they derive from waste material and can be useful in the perspective of a circular economy. These data agree with the micromorphological observations on the leaves from the two species, showing a lower gland density in EG with respect to that in EP. Figure 6A,B shows the chromatograms of the two EOs. The EOs composition with retention indices and area percentages for each compound are reported in Table 2; the compounds are listed according to their elution order on a HP-5MS column. Altogether, 37 compounds were identified, 30 for EG, accounting for 98.1% of the total EO, and 25 for EP, accounting for 97.7% of the total EO. The oxygenated monoterpenes are the main constituents in both EOs, with a percentage of 80.3% for EG and 79.1% for EP.

1,8-Cineole was the main constituent in both EOs, with a percentage of 74.7% in EG and 75.5% in EP. Other components present in a lesser amount in EG were α-pinene (13.1%) and terpineol (4.2%), while in EP, they were dihydro-eugenol (7.5%) and α-pinene (4.8%).

1,8-Cineole was reported as the main constituent of EG EO also in the samples from Argentina (17.9%); Serbia (67.8%); and Sardinia, Italy (33.0%), even if with a different percentage on total EO [33,34,35]. Regarding the other constituents, the Argentinian EO presented high percentages of *p*-cymene (12.3%), spathulenol (12.3%), and α-phellandrene (7.0%) [33], as well as from the Sardinian EO that showed high amounts of trans-sabinene hydrate acetate (15%), globulol (10.3%), and longicyclene (9.1%) [35]. Only the EO from Serbia presented α-pinene as the second major compound with a percentage of 14.1%, similar to that of the sample here presented [34]. Elaissi and coworkers [36] reported a Tunisian EG EO with a higher percentage of oxygenated sesquiterpenes (spathulenol, globulol, and viridiflorol) with respect to EO here reported, rich in oxygenated monoterpenes.

Few studies reported the chemical composition of EP EO [37,38,39]. The chemical composition of our sample agrees with those reported in these previous studies: 1,8-cineole was the main constituent, ranging from 75.1 to 85.1%, and α-pinene was present in comparable amounts with respect to our EO, ranging from 2.1 to 4.0%. However, there were also some differences; for example, α-terpinyl acetate was present in EOs from Tuscany (Italy) and Australia, but it was totally absent in the EO studied [37,39] and dihydro-eugenol, present in our sample, was absent in the EP EO from Morocco, Australia, and Tuscany [37,38,39]. An interesting study, which compared the chemistry and bioactivity of *E. globulus* EOs obtained by different extraction methods, concluded that there are some significant differences between EOs both in terms of high total yield and high concentration of volatiles [40]. Thus, the bioactivity of the EOs is also completely different and could be employed in different applications. This study can be a starting point for new research aimed at comparing different ways of obtaining EOs and selecting the most productive ones in the circular economy. In this way, it could be possible to implement these EO applications as bioherbicides, increasing the efficiency, soil persistency, and so on.

### 2.3. Phytotoxic and Anti-α-Amylase Activity

The possible phytotoxic activities of our EOs were tested on both weed (*Lolium multiflorum* Lam., *Portulaca oleracea* L.) and crop (*Raphanus sativus* L., *Lactuca sativa* L., *Lepidium sativum* L., *Solanum lycopersicum* L., *Pisum sativum* L., *Cucumis sativus* L.) plant species, in order to investigate both the possible herbicidal activity against weeds and the possible negative impact on crop growth.

Table 3 and Table 4 show the results of phytotoxic activity of the selected EOs against all seeds tested.

**Figure 7:**
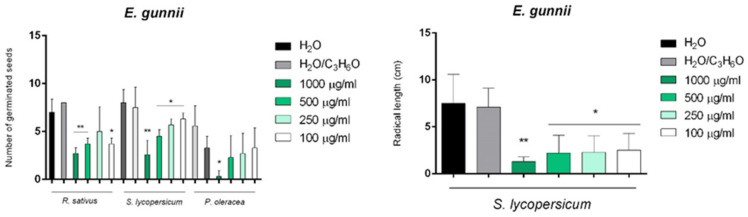
Phytotoxic activity of *E. gunnii* (EG) EO against the germination of *P. oleracea*, *R. sativus*, and *S. lycopersicum* and the radical elongation of *S. lycopersicum* 120 h after sowing. The results are the mean of three experiments ± standard deviation. * *p* < 0.05 and ** *p* < 0.01 compared with control (ANOVA followed by Dunnett’s multiple comparison test).

EG EO was able to inhibit the germination of *P. oleracea*, *R. sativus*, and *S. lycopersicum* seeds, while affecting only radical elongation in *S. lycopersicum* seeds (Figure 7).

**Figure 8:**
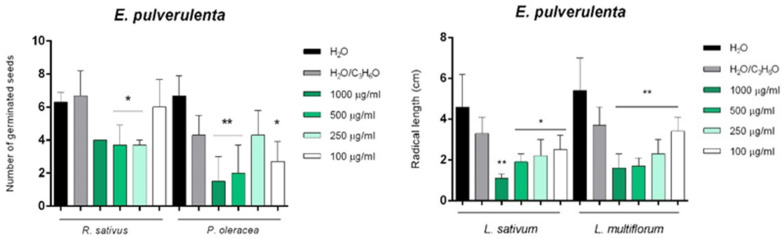
Phytotoxic activity of *E. pulverulenta* ‘Baby blue’ (EP) EO against germination of *P. oleracea* and *R. sativus*, and radical elongation of *L. sativum* and *L. multiflorum* 120 h after sowing. The results are the mean of three experiments ± standard deviation. * *p* < 0.05 and ** *p* < 0.01 compared with control (ANOVA followed by Dunnett’s multiple comparison test).

Instead, EP EO inhibited the germination of *P. oleracea* and *R. sativus*, and affected the radical elongation of *L. sativum* and *L. multiflorum* (Figure 8).

Moreover, the investigated EOs did not show any effects against the germination and/or radical elongation of *L. sativa*, *P. sativum*, and *C. sativus*.

Different species of *Eucalyptus* genus were studied for their allelopathic properties both against weeds and crops: *E. citriodora* Hook. was phytotoxic to *Bidens pilosa* L., *Amaranthus viridis* L., *Rumex nepalensis* Spreng., and *Leucaena leucocephala* (Lam.) de Wit, and caused injuries to *Triticum aestivum* L., *Zea mays* L., *Raphanus sativus* L., and *Oryza sativa* L. [23]; the EO from *E. nicholii* Maiden and Blakely strongly inhibited the germination of *Amaranthus retroflexus* L., *Portulaca oleracea* L., *Acroptilon repens* (L.) DC. [41], and *E. tereticornis* Sm. EO possessed allelopathic potential against *A. viridis* [42]. Rasaeifar and coworkers [16] reported that essential oil of *E. globulus* Labill. had poisonous effects on important weeds, such as *Amaranthus blitoides* S.Watson and *Cynodon dactylon* L., suggesting further research into the involved mechanisms to allow its use in cultivated lands. A lemon-scented *Eucalyptus* showed high phytotoxicity and its use as a bioherbicide has been proposed [22]. *Eucalyptus* EOs have been studied for the control of the noxious weed, *Parthenium hystorophorus* L. [43]. *E. globulus* EO, with a high content of 1,8-cineole, significantly affected seedling growth, showing a post-emergent herbicidal activity in *Echinochloa crus-galli* (L.) P. Beauv. [44]. The volatile cineoles are recognized as allelopathic agents [45].

Another common terpenoid present in EOs from many *Eucalyptus* species, α-pinene, played a key-role in root cell membranes’ disruption [24,25]. Even eugenol possessed a weed-suppressing ability, negatively affecting the photosynthetic efficiency and the energy metabolism of different weed species [19]. Moreover, monoterpenes have been proposed for their pivotal role in *Eucalyptus* communities [46].

Even though volatile allelochemicals derived from *Eucalyptus* EOs are probably among the most investigated for their herbicidal properties, no studies are currently available on the possible phytotoxic activity of EP EO. Regarding EG, our previous study on EO from Sardinia showed no phytotoxic activity against several tested seeds [35].

α-Amylase is a key enzyme involved in seed growth regulation; in fact, it hydrolyses the starch during the seed germination process [47]. Consequently, after the determination of the phytotoxic activity of the EOs, their possible activity on α-amylase regulation was studied. The results reported in Table 5 showed that the EP EO, with an IC_50_ value of 35.9 µg/mL, was more active than EG EO and acarbose, used as positive control.

In light of these results, it is possible to hypothesize that the variability in EOs phytotoxic activities may be related to the difference in the starch reserve of the seeds of the considered species. No previous studies reported the activity of EOs from *Eucalyptus* sp. pl. on α-amylase in association with phytotoxic activity.

### 2.4. Toxicity

The eco-compatibility of EG and EP EOs was evaluated using the toxicological test on *Artemia salina*, a marine zooplanktonic organism, which represents the gold standard in toxicological assays, owing to its easy cultivation, availability, low cost, and adaptation to adverse conditions [48]. Both EOs were tested in a wide dose range (0.01–100 mg/mL).

Potassium bichromate, used as positive control, inhibited the nauplii vitality after 24 and 48 h by 90 and 98%, respectively, showing a strong toxicity. The tested EOs did not show any toxicity or alteration of the nauplii swimming behaviour at 24 and 48 h until 10 mg/mL, with any statistically significant differences with respect to the negative control (data not shown). On the contrary, a mild toxicity at 24 h (16.66 vs. 13.33% for EG and EP EOs, respectively), which became strong at 48 h (85.0 vs. 50% for EG and EP EOs, respectively), was observed at the highest dose (100 mg/mL) for both EOs, with a statistically significant difference between them (*p* < 0.001, data not shown).

This is the first study to investigate the toxicity of EG and EP EOs on *Artemia salina* and no data are currently available on the eco-compatibility of these EOs.

However, already from these preliminary data, it is possible to speculate that both *Eucalyptus* EOs tested are safe in a wide dose range (0.01–10 mg/mL) for aquatic organisms and that they could be used safely in agriculture as eco-sustainable bio-herbicides.

## 3. Materials and Methods

### 3.1. Standards and Reagents

Soluble starch, PPA (porcine pancreatic α-amylase), HCl, KI, I_2_, K_2_Cr_2_O_7_, acetone, and acarbose were purchased from Sigma Aldrich (Milan, Italy).

### 3.2. Plant Material

*E. gunnii* (EG) and *E. pulverulenta* ‘Baby blue’ (EP) were collected in January 2021 in commercial plantations in the hinterland of Western Liguria (Finale Ligure, Savona, Italy) in Tovo San Giacomo (coordinates = 44.19683185° N, 8.26007775° E) and in Magliolo (coordinates = 44.1972954° N, 8.2558821° E), respectively. A voucher specimen of each species (GE5187 and GE5188, respectively) was deposited in the herbarium of the Department of Earth, Environment and Life Sciences, University of Genoa, Italy. The plants were approximately 10 months old and about 3 m high at the time of collection. About 1 kg of branches bearing juvenile foliage of each species was collected for both micromorphological analyses and EOs’ extraction.

### 3.3. Extraction of Essential Oils

The branches bearing juvenile foliage were reduced to small pieces and then subjected to hydrodistillation for 3 h, according to the standard procedure described in the European Pharmacopoeia [49]. The hydrodistillation of the aerial parts of EG and EP furnished pale yellow EOs at a yield of 1.1 and 1.6% on a dry mass basis, respectively. The EOs were dried over anhydrous sodium sulphate and kept under N_2_ at 4 °C in the dark, until analysis.

### 3.4. Micromorfological Analyses

For each species, eight leaves were selected in sequence along a branch, starting from the first fully expanded juvenile leaf, for micromorphological analyses. Two leaves for species were used to obtain epidermal peelings using the nail polish technique [50]. Six images of the imprints were captured at 10× magnification, both for the abaxial and adaxial epidermal surfaces, avoiding the central rib. The observations were performed in fields with the area corresponding to 0.930 mm^2^ using a Leica D.M. 2000 microscope equipped with a digital camera (DFC 320, Leica Microsystems, Wetzlar, Germany). The oil gland density was obtained according to the overlying cells on the epidermal surfaces, in agreement with the method previously described by Santos and coworkers [28]. For the histochemical analysis, another three leaves of each species were fixed, dehydrated, and paraffin embedded. Eight-micron thick cross sections were obtained using an automatic advance rotative microtome (Leica RM 2255, Leica Biosystems, Heidelberg, Germany) and stained with Toluidine Blue pH 4.0 [51] for anatomical and histological characterization. Microphotographs were taken using a Leica DMRB light microscope with a Leica CCD camera DFC420C (Leica, Switzerland).

Other leaf samples of both species were also processed for SEM analyses. Three portions for each species (about 1.5–2.0 cm^2^) were fixed in 70% ethanol-FineFix working solution (Milestone s.r.l., Bergamo, Italy) for 24 h at 4 °C, dehydrated through ethanol series [52], and then critical point dried in CO_2_ (CPD, K850 2M Strumenti s.r.l., Rome, Italy). Finally, the samples were mounted on the aluminium stubs using glued carbon tabs, sputter-coated with 10 nm gold [53], and observed with a Vega3 Tescan LMU SEM (Tescan USA Inc., Cranberry Twp, PA, USA) at an accelerating voltage of 20 kV. For each species, six images for both abaxial and adaxial surfaces were captured at 200× magnification to determine oil gland density.

All the captured images were analysed using image processing software ImageJ [54], which enables measurements and counting, allowing to obtain quantitative data.

### 3.5. GC-FID Analysis

Analytical gas chromatography (GC) was carried out on a Perkin-Elmer Sigma-115 gas chromatograph (Perkin Elmer, Waltham, MA, USA) equipped with a flame ionization detector (FID) and a data handling processor. The separation was achieved using a HP-5 MS fused-silica capillary column (30 m × 0.25 mm i.d., 0.25 μm film thickness, Agilent, Roma, Italy). Column temperature: 40 °C, with 5 min initial hold, and then to 270 °C at 2 °C/min, 270 °C (20 min); injection mode, splitless (1 μL of a 1:1000 *n*-hexane solution). Injector and detector temperatures were 250 °C and 290 °C, respectively. The analysis was also run using a fused silica HP Innowax polyethylene glycol capillary column (50 m × 0.20 mm i.d., 0.25 μm film thickness, Agilent, Roma, Italy). In both cases, helium was used as the carrier gas (1.0 mL/min).

### 3.6. GC/MS Analysis

Analysis was performed on an Agilent 6850 Ser. II apparatus (Agilent, Roma, Italy), fitted with a fused silica DB-5 capillary column (30 m × 0.25 mm i.d., 0.33 μm film thickness, Agilent, Roma, Italy), coupled to an Agilent Mass Selective Detector MSD 5973; ionization energy voltage 70 eV; electron multiplier voltage energy 2000 V. Mass spectra (MS) were scanned in the range 40–500 amu, scan time 5 scans/s. Gas chromatographic conditions were as reported in the previous paragraph; transfer line temperature, 295 °C.

### 3.7. Identification of the Essential Oil Components

Most constituents were identified by GC by comparison of their Kovats retention indices (Ri) (determined relative to the retention times (tR) of n-alkanes (C_10_–C_35_)), with either those of the literature [55,56,57,58] and mass spectra on both columns or those of authentic compounds available in our laboratories by means of NIST 02 and Wiley 275 libraries [59]. The component relative concentrations were obtained by peak area normalization. No response factors were calculated.

### 3.8. Phytotoxic Activity

The phytotoxic activity was evaluated on germination and radical elongation of several weed and crop species: *Raphanus sativus* L., *Lactuca sativa* L., *Lepidium sativum* L., *Solanum lycopersicum* L., *Pisum sativum L., Cucumis sativus L.,* *Lolium multiflorum* Lam., and *Portulaca oleracea* L. These seeds are often used for their easy and well-known germinability. *R. sativus*, *L. sativa*, *L. sativum*, *S. lycopersicum, P. sativum,* and *C. sativus* seeds were purchased from Blumen group SRL (Emilia-Romagna, Bologna, Italy). *L. multiflorum* seeds were purchased from Fratelli Ingegnoli Spa (Milano, Italy), and *P. oleracea* seeds from W. Legutko SRL (Jutrosin, Poland). The seeds were sterilized in 95% ethanol for 15 s and sown in Petri dishes (Ø = 90 mm), on three layers of Whatman filter paper. They were impregnated with 7 mL of deionized water used as first control to verify the germinability of the seeds, 7 mL of a water–acetone mixture (99.5:0.5, *v*/*v*) as second control because EOs were dissolved in this mixture for their lipophilicity, or 7 mL of the tested solution at different doses (1000, 500, 250, and 100 mg/mL). Controls, carried out with the water–acetone mixture alone, showed no appreciable differences in comparison with control in water alone. The germination conditions were 20 ± 1 °C, with a natural photoperiod. Seed germination was observed in Petri dishes every 24 h. A seed was considered germinated when the protrusion of the root became evident [60]. On the fifth day (after 120 h) for *R. sativus* and on the tenth day (after 240 h) for the other tested seeds, the effects on radicle elongation were measured in cm. Each determination was repeated three times, using Petri dishes containing 10 seeds each. Data are expressed as the mean ± standard deviation for both germination and radicle elongation.

### 3.9. α-Amylase Inhibitory Assay

Screening of plant material for α-amylase inhibition was carried out according to Xiao and coworkers [61] and Sudha and coworkers [62], based on the starch-iodine test with some modification. The total assay mixture composed of 200 μL 0.02 M sodium phosphate buffer pH 6.9 containing 2 units/mL of PPA, and EOs at a dose from 0.01 to 1.0 mg/mL (*w*/*v*) were incubated at 37 °C for 10 min. Then, soluble starch (0.5%, *w*/*v*) was added to each reaction cuvette and incubated at 37 °C for 15 min. Then, 1 M HCl (100 μL) was added to stop the enzymatic reaction, followed by the addition of 400 μL of iodine reagent (5 mM I_2_ and 5 mM KI). The colour change was recorded, and the absorbance was read at 620 nm on a microplate reader. The control reaction representing 100% enzyme activity did not contain any EO. The known PPA inhibitor, acarbose, was used as a positive control at a concentration range of 0.01–1 mg/mL. Finally, the α-amylase inhibition activity was calculated with the following formula:

$$\alpha -amylase\;inhibition\;activity\;\left(\%\right)=\frac{Abs_{1}}{Abs_{2}}\;100$$

where *Abs_1_* is the absorbance of sample solution mixed with amylase solution and soluble starch solution, and *Abs_2_* is the absorbance of the control (sample solution mixed with soluble starch solution). The IC_50_ values were defined as the dose of the EO, containing the α-amylase inhibitor, that inhibited 50% of the PPA activity.

### 3.10. Brine Shrimp Lethality Assay

In order to investigate the toxicity of the EG and EP EOs, the brine shrimp (*Artemia salina*) lethality assay was carried out according to Caputo and coworkers [35]. Brine shrimp’s eggs were purchased by a fish shop, placed in a hatcher chamber containing seawater, and incubated for 48 h at room temperature with continuous aeration and illumination. Stock solutions (0.01–100 g/mL) of EG and EP EOs, as well as potassium bichromate (K_2_Cr_2_O_7_, 50 mg/mL) used as a positive control, were prepared in DMSO and water, respectively. After that, 2 µL of each sample and control were seeded in a 24-well plate, and diluted 1:1000 *v*/*v* in seawater in order to achieve a 0.1% DMSO non-toxic concentration. Ten nauplii per well were added and incubated for 48 h under the same conditions reported above. Surviving larvae without abnormal swimming behavior after 24 h and 48 h were counted by a stereomicroscope (SMZ-171 Series, Motic Asia, Hong Kong, China). A negative control (ten larvae treated with 0.1% DMSO in seawater) was evaluated. Three independent experiments (*n* = 10) were carried out for each treatment. Lethality was calculated using the following equation:

% *Lethality* = 100 − [(*slt* × 100)] / *slcs*

where slt are the survival larvae treated with the extracts or K_2_Cr_2_O_7_, and slcs are the survival larvae treated with seawater (negative control).

## 4. Conclusions

Our study investigated the micromorphology, phytochemistry, phytotoxic activity, and eco-compatibility of *E. gunnii* (EG) and *E. pulverulenta* ‘Baby blue’ (EP) EOs. The comparison between the two species showed that EP had a higher oil gland density as well as a higher yield in EO with respect to EG. In both EOs, 1.8-cineole was the major compound (~75%); α-pinene was the second most abundant component in EG, while eugenol was the second in EP. Several studies have shown the phytotoxic activities of the EOs rich in terpenes, often linked to a high concentration of the afore-mentioned compounds. Our research highlighted the potential phytotoxicity of the EG and EP EOs against several weeds: both EOs inhibited *Portulaca oleracea* seed germination and EP EO also inhibited *Lolium multiflorum* radical elongation. Concerning crop species, the EOs investigated showed no effect against germination and/or radical growing of *Lactuca sativa,* *Pisum sativum,* and *Cucumis sativus*. On the contrary, EOs tested showed phytotoxicity on *Solanum lycopersicum*, *Lepidium sativum*, and *Raphanus sativus.* Our study also attested the inhibitory effects on a key enzyme involved in seed growth regulation, α-amylase; EP EO was much more active than EG EO.

The data collected show that the allelopathic action mechanisms of these EOs are various and they can be used as selective bioherbicides on target species. In addition, the high eco-compatibility in a wide dose range of the two EOs studied suggests their possible safe applications in agriculture. Finally, we point out the potential of remnants from *Eucalyptus* pruning for their transformation into value-added products for sustainable agriculture programs. In fact, weed control with allelopathic compounds released from crop residues is today regarded as a safe strategy in crop management systems in the frame of the circular economy, and by-products rich in essential oils play a primary role in this context.

## Figures and Tables

**Table 1:** *E. gunnii* (EG) and *E. pulverulenta* ‘Baby blue’ (EP) oil gland mean density (glands cm^−2^) and mean dimensions (μm).

Species	Oil Gland Mean Density (Glands cm^−2^) (±S.D.)		Oil Gland Mean Dimensions (μm) (±S.D.)	
	**Abaxial**	**Adaxial**	**Height**	**Width**
*E. gunnii*	213 (±66)	416 (±116)	199 (±34)	161 (±31)
*E. pulverulenta*	750 (±96)	1321 (±176)	188 (±32)	142 (±26)

The data of gland density and dimensions are expressed as mean ±S.D. Oil gland density. EG: abaxial vs. adaxial, *p* < 0.01, *n* = 12; EP: abaxial vs. adaxial, *p* < 0.01, *n* = 12; EG: abaxial vs. EP abaxial, *p* < 0.01, *n* = 12: EG: adaxial vs. EP adaxial, *p* < 0.01, *n* = 12. Oil gland dimensions. EG vs. EP height and width, *p* > 0.05, *n* = 21. *t*-test was used for comparisons of the micromorphological data. The results were considered significant for *p* < 0.01.

**Table 2:** Chemical composition of *E. gunnii* (EG) and *E. pulverulenta* ‘Baby blue’ (EP) EOs.

	RT	Compound	%		KI ^a^	KI ^b^	Identification ^c^
			EG	EP			
1	5.148	1-Methyl-1,3-cyclohexadiene		t	759	1183	1,2
2	5.267	3-Methyl-2-buten-1-ol		t	761	1328	1,2
3	6.246	*n*-Octane		t	773		1,2
4	7.299	(2Z)-Hexenol		t	787		1,2
5	12.004	Santolina triene	t		848	1043	1,2
6	13.849	α-Pinene	13.1	4.8	872	1028	1,2,3
7	14.036	Camphene	0.1	0.1	874	1075	1,2,3
8	16.071	β-Pinene	0.3	0.5	901	1120	1,2,3
9	16.945	dehydro-1,8 Cineole	t		912	1095	1,2
10	17.952	Myrcene	0.3	0.4	925	1173	1,2,3
11	18.695	δ-2-Carene	0.1	0.2	934	1146	1,2,3
12	19.400	β-Phellandrene		t	944	1206	1,2,3
13	19.500	*p*-Cymene	t		945	1279	1,2,3
14	20.574	1,8-Cineole	74.7	75.5	959	1220	1,2,3
15	21.870	(Z)-β-Ocimene	t		976	1240	1,2,3
16	22.438	(E)-β- Ocimene	1.9	1.0	983	1260	1,2,3
17	25.243	γ-Terpinene	0.3	0.9	1016	1254	1,2,3
18	27.271	dehydro-Linalool	0.5	0.5	1043		1,2
19	27.644	1,3,8-*p*-Menthatriene	0.4		1048		1,2
20	28.128	*allo*-Ocimene	0.1		1055	1382	1,2
21	28.799	*neo*-Isopulegol	t		1064		1,2
22	29.513	Borneol	0.2		1074	1715	1,2,3
23	29.776	*endo*-Fenchol	0.2	1.0	1077		1,2
24	31.870	Terpineol	4.2	1.5	1099	1710	1,2,3
25	32.268	*cis*-Verbenol	0.2	0.1	1104	1663	1,2
26	34.118	Verbenone	0.2	0.2	1131		1,2
27	39.679	Thymol	0.1	0.3	1205	2172	1,2,3
28	40.403	δ-Elemene	0.1		1216	1460	1,2,3
29	41.032	γ-Elemene	0.1		1226	1651	1,2,3
30	41.512	Myltayl-4(12)-ene	0.1		1233		1,2
31	42.079	dihydro-Eugenol	0.5	7.5	1242		1,2
32	43.444	α-Copaene	t	0.1	1262	1477	1,2,3
33	45.520	Sibirene	t	0.7	1294		1,2
34	46.066	Caryophyllene	0.2		1296	1607	1,2,3
35	50.831	α-Guaiene	0.2	0.3	1372	1583	1,2
36	52.204	Cycloisolongifol-5-ol <trans>		0.2	1395		1,2
37	56.184	Globulol		1.9	1457	2095	1,2
		Total	98.1	97.7			
		Monoterpene hydrocarbons	17.1	15.4			
		Oxygenated monoterpenes	80.3	79.1			
		Sesquiterpenes hydrocarbons	0.7	1.1			
		Oxygenated sesquiterpenes		2.1			

^a, b^ The Kovats retention indices determined relative to a series of n-alkanes (C_10_–C_35_) on the apolar HP-5 MS and the polar HP Innowax capillary columns, respectively; ^c^ identification method: 1 = comparison of the Kovats retention indices with published data, 2 = comparison of mass spectra with those listed in the NIST 02 and Wiley 275 libraries and with published data, and 3 = coinjection with authentic compounds; t = trace (<0.1%).

**Table 3:** Inhibition of germination of *L. sativa, P. oleracea, R.sativus, S. lycopersicum, P. sativum, C. sativus, L. sativum,* and *L. multiflorum* seeds treated with different doses of EOs. Results are reported as the mean ± SD of three experiments. * *p* <0.05, ** *p* <0.01, *vs*. control (inhibition = 0), according to two way ANOVA followed by Tuckey’s multiple comparison test, at the significance level of *p* < 0.05.

Seeds		*E. gunnii* EO (µg/mL)				*E. pulverulenta* EO (µg/mL)			
		1000	500	250	100	1000	500	250	100
*L. sativa*	Mean ± SD	7.7 ± 0.6	8.3 ± 0.6	8.0 ± 0.0	9.5 ± 0.7	9.0 ± 0.0	8.0 ± 0.0	8.5 ± 0.7	8.5 ± 0.7
	%	9.4	2.3	0	0	0	5.9	0	0
*P. oleracea*	Mean ± SD	0.3 ± 0.6 *	2.3 ± 2.3	2.7 ± 2.1	3.3 ± 2.1	1.7 ± 1.5 **	2.0 ± 1.7 **	4.3 ± 1.5	2.7 ± 1.2 *
	%	94.6	58.9	51.8	41.1	74.7	70.2	35.9	59.8
*R. sativus*	Mean ± SD	2.7 ± 0.6 **	3.7 ± 0.6 *	5.0 ± 2.6	3.7 ± 0.6 *	4.0 ± 0.0	3.7 ± 1.2 *	3.7 ± 1.5 *	6.0 ± 1.7
	%	61.5	47.2	28.6	47.8	36.6	41.3	41.3	38.4
*S. lycopersicum*	Mean ± SD	2.6 ± 1.5 **	4.5 ± 0.7 *	5.7 ± 0.6	5.6 ± 0.6	8.3 ± 0.6	7.3 ± 0.6	7.3 ± 0.6	8.2 ± 0.6
	%	67.5	43.8	27.8	30	0	8.7	8.7	0
*P. sativum*	Mean ± SD	9.0 ± 1.7	9.0 ± 1.7	7.3 ± 2.1	9.3 ± 1.2	9.3 ± 0.6	8.7 ± 0.6	10 ± 0.0	9.7 ± 0.6
	%	7.2	7.2	24.7	4.1	4.1	10.3	3	0
*C. sativus*	Mean ± SD	6.0 ± 0.0	5.3 ± 0.6	5.7 ± 0.6	4.3 ± 0.6	5.3 ± 0.6	5.7 ± 0.6	6.0 ± 0.0	6.3 ± 0.6
	%	25	33.7	28.7	46.2	11.6	5	0	0
*L. sativum*	Mean ± SD	9.0 ± 1.0	8.3 ± 0.6	7.5 ±0.7	9.1 ± 0.3	7.0 ± 0.0	6.7 ± 1.5	6.7 ± 2.1	8.0 ± 1.7
	%	5.5	2.3	11.8	0	12.5	16.2	16.2	0
*L. multiflorum*	Mean ± SD	7.0 ± 2.0	7.5 ± 0.7	7.7 ± 0.6	7.0 ± 2.6	7.3 ± 0.6	6.0 ± 1.0	7.0 ± 0.0	8.3 ± 0.6
	%	12.5	6.2	3.7	12.5	0	14.2	0	0

**Table 4:** Inhibition of radical elongation of *L. sativa, P. oleracea, R.sativus, S. lycopersicum, P. sativum, C. sativus, L. sativum*, and *L. multiflorum* seeds treated with different doses. Results are reported as the mean ± SD of three experiments. * *p* <0.05, ** *p* <0.01, *vs*. control (inhibition = 0), according to two way ANOVA followed by Tuckey’s multiple comparison test, at the significance level of *p* < 0.05.

Seeds		*E. gunnii* EO (µg/mL)				*E. pulverulenta* EO (µg/mL)			
		1000	500	250	100	1000	500	250	100
*L. sativa*	Mean ± SD	1.7 ± 0.6	2.5 ± 0.5	2.3 ± 0.6	2.6 ± 0.7	2.2 ± 0.5	2.1 ± 0.3	2.2 ± 0.5	2.1 ± 0.5
	%	37.0	7.4	17	3	4.5	9.5	4.5	9.5
*P. oleracea*	Mean ± SD	2.3 ± 0.4	2.1 ± 0.2	2.2 ± 0.3	2.0 ± 0.1	1.9 ± 0.4	2.1 ± 0.3	2.3 ± 0.2	2.2 ± 0.2
	%	0	0	0	0	0	0	0	0
*R. sativus*	Mean ± SD	1.0 ± 0.2	1.2 ± 0.6	1.5 ± 0.3	1.7 ± 0.4	1.8 ± 0.5	1.5 ± 0.8	1.7 ± 0.3	2.4 ± 0.6
	%	0	0	0	0	0	0	0	0
*S. lycopersicum*	Mean ± SD	1.3 ± 0.5 **	2.2 ± 1.9 *	2.3 ± 1.7 *	2.5 ± 1.8 *	3.2 ± 1	4.5 ± 1.7	4.6 ± 1.7	5.8 ± 1.8
	%	82.6	70.7	69.3	66.7	57.3	40	38.7	22.7
*P. sativum*	Mean ± SD	4.1 ± 1.0	4.0 ± 1.0	4.5 ± 1.1	3.9 ± 0.9	3.9 ± 0.6	3.7 ± 0.8	4.5 ± 1.1	4.8 ± 0.9
	%	0	0	0	0	0	0	0	0
*C. sativus*	Mean ± SD	6.0 ± 1.6	7.0 ± 1.0	6.9 ± 1.7	6.4 ± 0.8	5.4 ± 0.7	7.5 ± 0.9	6.7 ± 1.1	7.2 ± 1.9
	%	15.5	1.4	2.8	9.9	23.9	0	5.6	0
*L. sativum*	Mean ± SD	2.0 ± 1.4	3.0 ± 1.2	3.2 ± 1.3	2.5 ± 1.8	1.1 ± 0.2 **	1.9 ± 0.4 *	2.2 ± 0.8 *	2.5 ± 0.7 *
	%	63.0	44.4	40.7	53.7	76.0	58.7	52.2	45.6
*L. multiflorum*	Mean ± SD	2.6 ± 0.6 *	3.5 ± 1.1	4.0 ± 1.0	3.5 ± 1.0	1.6 ± 0.7 **	1.7 ± 0.4 **	2.3 ± 0.7 **	3.4 ± 0.7
	%	53.5	37.5	28.6	37.5	70.4	68.5	57.4	37.0

**Table 5:** α-Amylase inhibitory activity of *E.gunnii* and *E. pulverulenta* ‘Baby blue’ EOs.

Essential Oil	IC_50_ (µg/mL)
*E. pulverulenta*	35.9 ± 3.6
*E. gunnii*	524.1 ± 10.3
Acarbose (positive control)	130.2 ± 12.4

Data are expressed as mean ±S.D. (*n* = 3).

## Data Availability

The data presented in this study are available on request from the corresponding author.

## Sample Availability

Samples of the *E. gunnii* and *E. pulverulenta* ‘Baby blue’ essential oils are available from the authors. Vouchers were deposited in the Herbarium of Department of Earth, Environment and Life Sciences, University of Genoa.
